# *Emblica* *officinalis* Extract Induces Autophagy and Inhibits Human Ovarian Cancer Cell Proliferation, Angiogenesis, Growth of Mouse Xenograft Tumors

**DOI:** 10.1371/journal.pone.0072748

**Published:** 2013-08-15

**Authors:** Alok De, Archana De, Chris Papasian, Shane Hentges, Snigdha Banerjee, Inamul Haque, Sushanta K. Banerjee

**Affiliations:** 1 Department of OB/GYN, School of Medicine, University of Missouri Kansas City, Kansas City, Missouri, United States of America; 2 Cancer Research Unit, VA Medical Center, Kansas City, Missouri, United States of America; 3 Department of Basic Medical Science, School of Medicine, University of Missouri Kansas City, Kansas City, Missouri, United States of America; 4 Department of Biomedical Sciences, Colorado State University, Fort Collins, Colorado, United States of America; 5 Division of Hematology and Oncology, Department of Medicine, University of Kansas Medical Center, Kansas City, Kansas, United States of America; 6 Department of Anatomy and Cell Biology, University of Kansas Medical Center, Kansas City, Kansas, United States of America; Baylor College of Medicine, United States of America

## Abstract

Patients with ovarian cancer (OC) may be treated with surgery, chemotherapy and/or radiation therapy, although none of these strategies are very effective. Several plant-based natural products/dietary supplements, including extracts from *Emblica* *officinalis* (Amla), have demonstrated potent anti-neoplastic properties. In this study we determined that Amla extract (AE) has anti-proliferative effects on OC cells under both *in vitro* and *in vivo* conditions. We also determined the anti-proliferative effects one of the components of AE, quercetin, on OC cells under *in vitro* conditions. AE did not induce apoptotic cell death, but did significantly increase the expression of the autophagic proteins beclin1 and LC3B-II under *in vitro* conditions. Quercetin also increased the expression of the autophagic proteins beclin1 and LC3B-II under *in vitro* conditions. AE also significantly reduced the expression of several angiogenic genes, including hypoxia-inducible factor 1α (HIF-1α) in OVCAR3 cells. AE acted synergistically with cisplatin to reduce cell proliferation and increase expression of the autophagic proteins beclin1 and LC3B-II under *in vitro* conditions. AE also had anti-proliferative effects and induced the expression of the autophagic proteins beclin1 and LC3B-II in mouse xenograft tumors. Additionally, AE reduced endothelial cell antigen – CD31 positive blood vessels and HIF-1α expression in mouse xenograft tumors. Together, these studies indicate that AE inhibits OC cell growth both *in vitro* and *in vivo* possibly via inhibition of angiogenesis and activation of autophagy in OC. Thus AE may prove useful as an alternative or adjunct therapeutic approach in helping to fight OC.

## Introduction

Ovarian cancer (OC) is the second most common gynecological cancer and is the leading cause of cancer death in women in the United States [1]. Each year approximately 22,000 women in United States are diagnosed with OC, and 15,000 deaths were attributable to OC in 2011 alone [1]. OC is difficult to diagnose at its early stages (I/II), and is often not clinically suspected until it spreads and advances to the later stages (III/IV). Consequently, OC has a poor prognosis, with a five year survival rate for all stages of ~ 47% [2]. Currently, OC may be treated with surgery, chemotherapy and radiation, with suboptimal results as indicated by the five year survival rate cited above. The development of new anticancer drugs, or combinations of drugs for OC have not provided significant reason to be optimistic. Since conventional anti-cancer drugs can be highly toxic, plant-derived bioactive compounds are being investigated more intensively as alternate or adjunct therapies for various forms of cancer [3]. Recent evidence suggests that plant extracts have anti-tumor/anti-cancer/anti-proliferative effects on cultured human tumor cell lines [4–7] and also have an antiangiogenic effect on cancer cell lines [8].

Amla ( *Emblica* *officinalis* ) is a fruited plant that has been recognized for its medicinal value, and has been used since ancient times in the Indian traditional system of medicine ‘Ayurveda’ for treating several diseases including cancer [9–11]. The fruit of the Amla plant contains 11 known medicinally relevant components such as gallic acid, ellagic acid, 1-O-galloyl-beta-D-glucose, 3,6-di-O-galloyl-D-glucose, chebulinic acid, quercetin, chebulagic acid, corilagin, 1,6-di-O-gallolyl beta D glucose, 3-ethylgallic acid, isostrictiniin and ascorbic acid [9]. The branches of this plant contain seven bioactive compounds – geraniin, phyllanemblinins C and E, prodelphinidin B1, (2)-epigallocatechin 3-O-gallate, (S)-eriodictyol 7-[6-O-(E)-p-coumaroyl]-b-D-glucoside [12]. From among this collection of compounds, gallic acid, ellagic acid, 1-O-galloyl-beta-D-glucose, chebulinic acid, quercetin, chebulagic acid, corilagin, ascorbic acid, and geraniin have demonstrated strong anti-carcinogenic properties individually, and these may help explain the anti-cancer properties of whole Amla extract (AE) [9,13]. AE has been shown to inhibit proliferation of a variety of cancer cells *in vitro*, including OC cells, and also has demonstrated anti-proliferative effects *in vivo* [9–11]. Recently triphala, an herbal remedy containing AE, has also demonstrated anti-angiogenesis properties [8].

Due to the potential value of AE as an anti-cancer therapy, particularly for OC, we have investigated the anti-proliferative and anti-tumorigenic effects of AE in ovarian cell lines *in vitro* and in a mouse xenograft model. We have also investigated the effect of AE on tumor angiogenesis in cultured cells and a mouse xenograft model. We have observed that AE did not induce apoptotic cell death, but did significantly increase the expression of the autophagic protein in tissue culture and mouse xenograft model. We have also observed that AE acted synergistically with cisplatin to reduce cell proliferation and increase expression of beclin1 and LC3B-II under *in vitro* conditions.

## Materials and Methods

### Ethics Statement

All the animals were maintained according to standard guidelines of American Association for the Accreditation of Laboratory Animal Care. The study was approved by the Institutional

Animal Care and Use Committee of the Kansas City VA Medical Center (Kansas City, MO).

### Cell culture and reagents

OVCAR3, SW626 and normal human placental cells (HS 799 pl) were obtained from the American Type Culture Collection. Dulbeccos Modified Eagle’s Medium (DMEM) and trypsin were purchased from Sigma, St. Louis, MO. OVCAR3 and SW626 cells were maintained in DMEM with 10% fetal bovine serum (Hyclone Laboratories, Logan, UT) and antibiotics at 37 ^O^C in a 5% CO_2_ environment. All cells used in this study were within 10 passages after receipt or recovery (~2 and 1/2 months of culturing). AE for treatment was prepared from commercially available tablets (Himalaya, USA, Houston, TX, containing 250 mg of Amla fruit and 350 mg of Amla stem powder). A stock solution of AE was prepared by weighing the Amla tablets, grinding them, and dissolving the powder in endotoxin free sterile water at 10 mg/ml. The solution was filtered through a 0.02 µm cellulose acetate membrane and used to treat the culture at different concentrations.

### Treatment

10,000 OVCAR3 or SW626 cells were plated in individual wells of 24-well plates in 1 ml medium containing 10% fetal bovine serum and incubated at 37 ^O^C for 24 h before the start of the experiments. After the initial plating, the medium was replaced with 1 ml of DMEM containing serum (10%) and AE (0-400 µg/ml; in triplicate) for various time points (6-96 hours) at 37 ^O^C. The *in vitro* control (0 µg/ml of AE) received vehicle (water) at a volume equal to the highest concentration of AE used. We also treated OVCAR3 cells with different doses of quercetin (5-100 µg/ml; in quadruplicate) for various time points (6-96 hours) at 37 ^O^C. The *in vitro* control (0 µg/ml of quercetin) received vehicle (DMSO; 4µl, which is equivalent to the volume for 100 µg/ml of quercetin).

### Cell proliferation

Cell proliferation was assessed using [3-(4,5-dimethylthiazol-2-yl)-2,5-diphenyltetrazolium bromide)] (MTT) assays as described previously [14] with modifications [15]. OVCAR3 and SW626 cells were grown in 24-well plates containing DMEM and 10% FBS. After treatment, MTT (0.1 mg/well) was added to cells followed by incubation for 4 h at 37 ^O^C. The formazan crystals formed were solubilized by incubating the cells with 1 ml of isopropyl alcohol which dissolved the blue formazon product that occurred during incubation with MTT. The optical density was measured at 560 nM in a spectrophotometer. The number of functionally active cells (i.e., optical density values) was calculated for comparisons between control and treated groups.

### DNA fragmentation

DNA was prepared from treated and untreated cultures as described previously [16]. Briefly, after treatment the cells were lysed in 100 µl of lysis buffer (100 mM Tris, pH 8.0, 20 mM EDTA, 0.5% SDS) treated with 50 µg/ml DNase-free RNase at 37 ^O^C for 30 min and 100 µg/ml proteinase K for 2 h at 50 ^O^C. The DNA was then extracted with phenol-chloroform and chloroform and precipitated in 3 volumes of ethanol. The DNA (1 µg/lane) was electrophoresed on 1% agarose gels. Molecular weight standards were run concurrently. The gels were stained with ethidium bromide and photographed using a Chemidox (BioRad, Hercules, CA).

### RNA Extraction

Total RNA was extracted from OVCAR3 cells using Trizol extraction method. mRNA quantity and quality were determined by measuring its absorbance in Genesys 6 Scanning UV/VIS Scanning Spectrophotometer and also using RNA experion chip (BioRad Laboratories, Hercules, CA, USA).

### Gene Array

Differential expression of angiogenesis regulatory genes was analyzed using Oligo GEArray provided by the manufacturer (SA Bioscience Corporation, Frederick, MD, USA). Briefly, 3µg total RNA was reverse transcribed into Biotin-16-dUTP-labelled cDNA probes with the TrueLabeling-AMP method using 16 µl 2.5X RNA polymerase, 2 µl 10 mM biotinylated UTP, RNA Polymerase 2 µl. The SuperArray membranes (OHS-026) were pre-hybridized at 60 ^O^C for 2 hours. Hybridization of the Biotin-labeled cDNA probes to the membranes was carried out at 60 ^O^C overnight with slow agitation. The hybridized membranes were washed in saline sodium citrate buffer (once in 2x SSC, 1% SCS and once in 0.1 x SSC, 0.5% SDS). The membranes were incubated with alkaline phosphatase-conjugated streptavidin, and then with the chemiluminescent substrate CDP-Star. Images of the membranes were acquired using the Chemidoc XRS system (BioRad) and the datasets were exported to GEArray Analyzer, the software developed by SA Bioscience and analyzed. The relative expression level of each gene was determined by comparing the signal intensity of each gene in the array after correction for background and normalization.

### In vivo tumor studies

Eight-week-old nude mice (nu/nu genotype, Harlan Laboratories (Madison, WI) were maintained with water and food *ad libitum* in a pathogen free environment with a 12 h light and 12 h dark cycle in an animal care facility at Kansas City VA Medical Center. Animal care and experimental procedures were performed according to the approved Guidelines of the Animal Care and Use Committee of Kansas City VA Medical Center. OVCAR3 cells (5X10^4^) with Matrigel were injected subcutaneously into the right rear flank of each mouse (4-5 mice per group). Tumor growth was monitored after the 2^nd^ day of injection and continued up to 24 days. Tumor length and width were measured using a caliper and the tumor volume was calculated using the formula: tumor volume = length x width x 0.5 width.

Six days after injection, mice were fed orally with 10% sucrose (control) or 10% sucrose with AE (100 mg/kg body wt.) daily. The dose 100 mg/kg body weight was chosen based on a preliminary study where this dose showed optimal effect on inhibition of tumor growth. After 18 days of AE treatment, mice were sacrificed and tumors were removed and fixed in 4% buffered formaldehyde for further study.

### Immunohistochemistry

Immunohistochemistry was performed on 4% formalin fixed-paraffin-embedded tissue sections according to our previous methods [17,18]. Briefly, tissue sections were fixed in 4% buffered formaldehyde and deparaffinized in xylene, rehydrated in descending concentrations of alcohol, washed with PBS and blocked with blocking serum (Immpress-Vector Laboratories, Burlingame, CA) for 20 minutes. The sections were incubated with beclin1, LC3B-II (Cell Signaling, Boston, MA), CD31, Ki67 (ABcam, Cambridge, MA) or Hif-1α (Novus Biologicals, Littleton CO) antibody (1:500 for all antibodies) overnight in a moist chamber at 4 ^O^C. The immunoreactivity was detected using secondary antibodies conjugated to streptavidin (Immpress-Vector Laboratories) and the sections were counterstained with hematoxylin. The sections were imaged with a Leica digital photomicroscope. For quantification of microvessels, tissue sections were analyzed at 50X magnification and CD31-positive vessels were counted. Four areas from each of 3 sections per tumor were analyzed.

OVCAR3 cells were fixed in 4% buffered formaldehyde and washed in PBS. After blocking in blocking serum, the cells were incubated with beclin1, LC3B-II or Hif-1α (1:200) antibody overnight at 4 ^O^C. The cells were washed and immunoreactivity was detected as described above. The number of immunostained cells and total cells were counted and the percentage of cells with immunolabeling was calculated.

### Western blot

Western blots were performed as previously described [19]. Briefly, the protein was extracted either from OVCAR3, SW626 cells or from tumors and the concentration of protein was measured by the BCA protein assay method (Pierce, Rockford, IL). Each sample (50 µg) was run on an SDS-sodium dodecyl sulfate-polyacrylamide gel. The protein was transferred to a nitrocellulose membrane. The membrane was incubated with blocking serum (Thermoscientific, Pittsburgh, PA) for 1 h at room temperature followed by overnight incubation at 4 ^O^C with antibody to Bax, Bcl2, Caspase 3, cleaved caspase 3, Caspase 7, beclin1 or LC3B-II (1:1000, Cell Signaling) or Hif-1α (1:1000, Abcam). After washing with Tris-NaCl-tween 20 buffer, the membrane was incubated with secondary antibodies (1:10000, Abcam) at room temperature for 1 h. Immunoreactivity was detected using enhanced chemiluminescence (Thermoscientic).

### Statistical analysis

Assays were performed in triplicate and each experiment was repeated twice. All graphical data are displayed as mean + S.E.M. Significance was tested using unpaired, two-tailed Student’s t-Tests with unequal variance (Microsoft Excel, Redmond, WA). *p* < 0.05 was considered significant.

## Results

### AE inhibits OVCAR3 and SW626 cells proliferation

**Figure 1:**
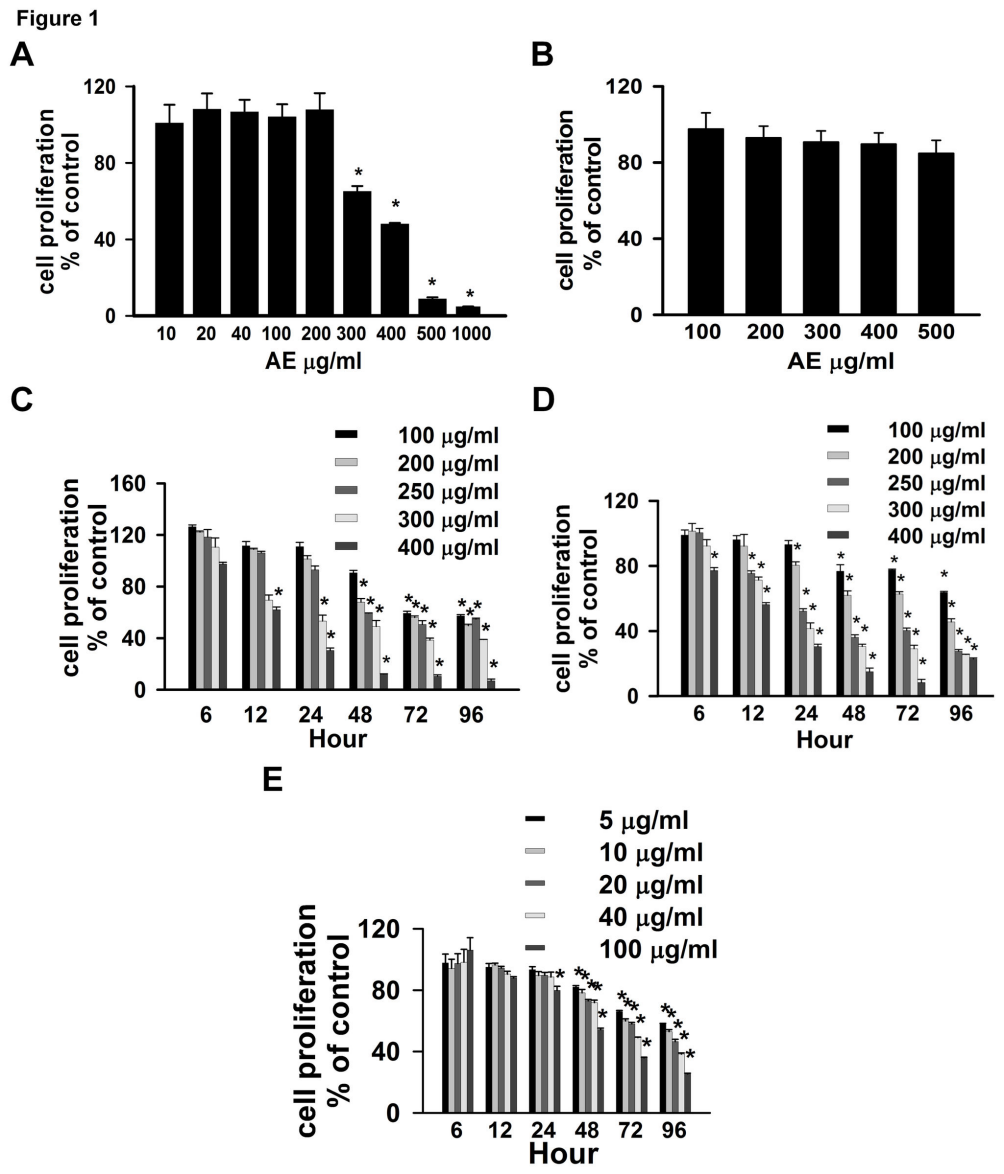
AE and quercetin treatment reduces cell proliferation and alters morphology of OC cells. A. Dose dependent effect of AE on proliferation of OVCAR3 cells. B. Dose dependent effect of AE on cell death in normal placental cells (HS-799 pl). C–D. Time and Dose dependent effects of AE on proliferation OVCAR3 (C) and SW626 (D) cells. E. Dose dependent effect of quercetin on proliferation of OVCAR3 cells. OVCAR3 and SW626 cells were cultured and grown for 2 days in DMEM in presence of 10% serum as described under Materials and Methods. After this period, the cultures were fed with medium containing 10% serum and different doses of AE for 24 hours except time dependent study. Data are the mean + S.E.M. from 6 independent observations. *, p<0.05, significantly different from vehicle-treated control group.

To determine if AE can affect the cell proliferation, we tested its effect on three different cell lines: 1) an ovarian cancer cell (OVCAR3), 2) a primary adenocarcinoma of the colon metastasized on ovary (SW626), 3) a normal human placental cells (HS 799). Normal placental cells were used to determine if AE has any effect on non-neoplastic cells. In addition, we used SW626 cells to determine if AE is able to inhibit tumors that had metastasized from other sites to the ovary, and to determine whether the effects of AE on OVCAR3 would be duplicated with other cancer cells. OVCAR3 cells were treated with varying concentrations of AE (0-1000 µg/ml) for 24 hour and the relative number of cells was inferred using MTT assays. Low concentrations (10-200 µg/ml) of AE did not affect cell proliferation; however, cell proliferation was significantly inhibited at AE concentrations ranging from 300–1000 µg/ml (Figure 1A). We treated normal placental cells (HS 799 pl) with different doses (100-500 µg/ml) of AE for 48 hour to determine if AE was generally cytotoxic, and determined that AE had essentially no impact on cell survival at any of the concentrations tested (Figure 1B). We treated both OVCAR3 and SW626 cells in culture with increasing concentrations of AE for various times prior to performing MTT assays. Rapid loss of cell proliferation occurred with increasing concentrations of AE in both OVCAR3 and SW626 cells (Figure 1A, 1C, 1D). In both cell lines, the inhibition of cell proliferation induced by AE was dependent on both concentration and incubation time (Figure 1C, 1D). The lowest concentration of AE tested (100 µg/ml) caused significant inhibition (p=<0.001) of OVCAR3 cells by 72 hour, and the highest dose (400 µg/ml) potently inhibited (p=<0.007) growth of OVCAR3 cells by 12 hour (Figure 1C). In SW626 cells, AE at 100 µg/ml caused significant inhibition by 48 hour (p=0.001), and at the highest dose growth was inhibited (p=<0.001) by 6 hour (Figure 1D).

### Quercetin inhibits cell proliferation in OVCAR3 cells

After determining that AE dose- and time-dependently reduced cell proliferation in OVCAR3 cells, we tested whether treatment of OC cells with different doses (0-100 µg/ml) of quercetin--one of the components of AE-- could reduce cell proliferation in OVCAR3 cells. The lowest concentration of quercetin tested (5 µg/ml) caused significant inhibition (p=<0.01) of OVCAR3 cells by 48 hr, and the highest dose (100 µg/ml) inhibited (p=<0.005) growth of OVCAR3 cells by 24 hr (Figure 1E).

### AE changes the morphology of OVCAR3 and SW626 cells

**Figure 2:**
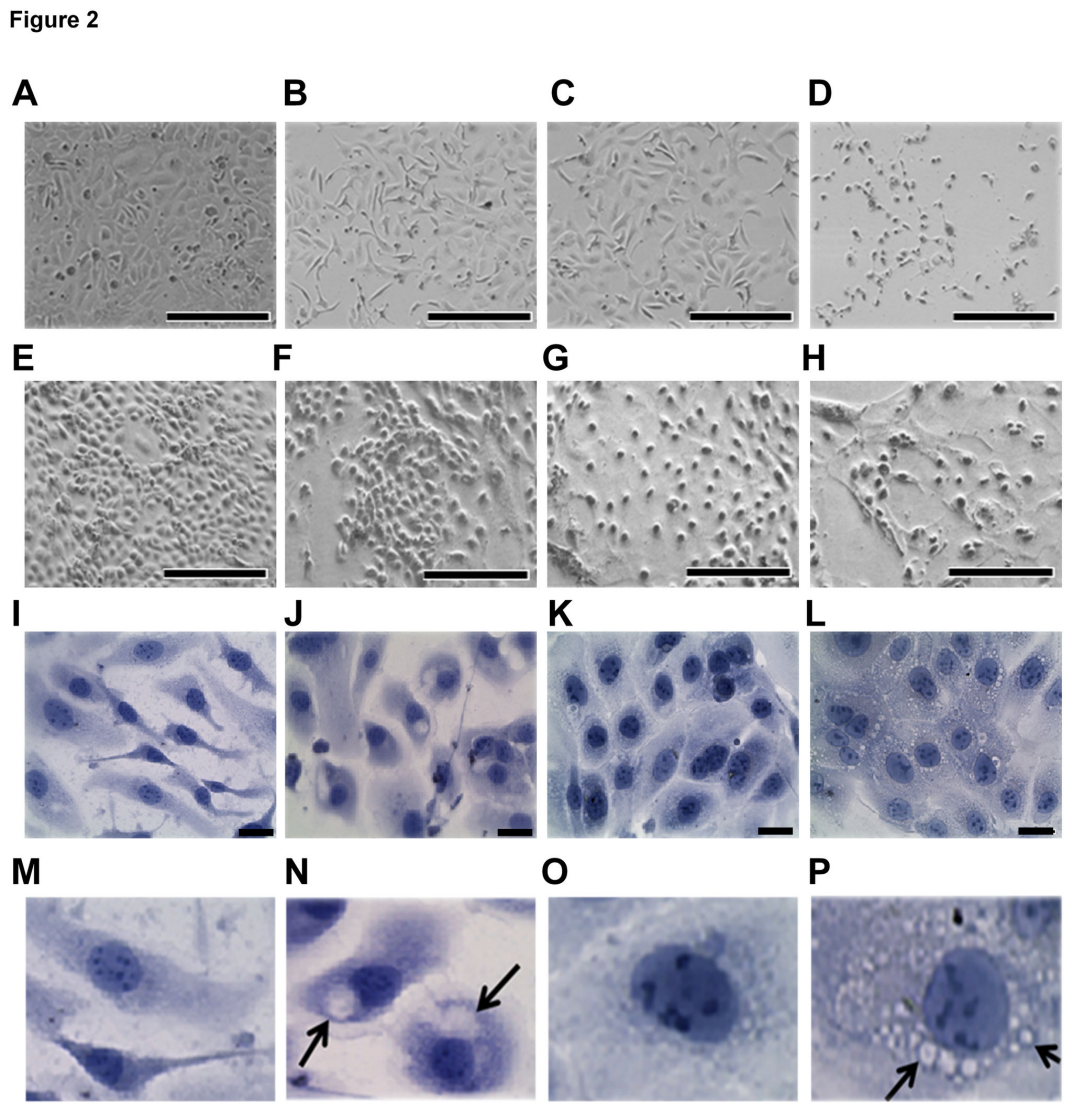
AE treatment alters morphology of OC cells. A–D. Dose dependent effect of AE on morphology and cell density of OVCAR3: A = Control, B=100 µg/ml AE, C=200 µg/ml AE, D=300 µg/ml AE. E–H. Dose dependent effect of AE on morphology and cell density of SW626 cells. E = Control, F=100 µg/ml AE, G=200 µg/ml AE, H=300 µg/ml AE. I–J, M–N. AE increased cytoplasmic vacuoles in OVCAR3. I and M = Control, J and N = 300 µg/ml AE. K–L and O–P. AE increased cytoplasmic vacuoles in SW626. K and O = Control, L and P=300 µg/ml AE. OVCAR3 and SW626 cells were cultured and grown for 2 days in DMEM in presence of 10% serum as described under Materials and Methods. After this period, the cultures were fed with medium 10% serum and different doses of AE for 24 hours. Some of the vacuoles are indicated by arrows (N and P). Bar=50 µm.

Inhibition of proliferation may change the size and morphology of the individual cells [20]. Therefore, we carefully observed the morphology of OVCAR3 and SW626 cells after treatment with 0-300 µg/ml AE. The morphology of OVCAR3 cells treated with 100 and 200 µg/ml AE was indistinguishable from untreated cells after 24 hour (Figure 2 A–C), whereas treatment with 300 µg/ml for 24 hour caused the majority of the cells to become round (Figure 2D). AE also induced dose dependent alterations in the morphology of SW626 cells after 24 hour (Figure 2E–H). Closer examination revealed that treatment with 300 µg/ml of AE for 24 hours induced cytoplasmic vacuoles in both OVCAR3 and SW626 cells (Figures 2J, 2N, 2L, 2P). These morphologic changes suggest that cell death may be occurring since cell death is sometimes accompanied by vacuole formation [21]. Further experiments were performed using 300 µg/ml concentration of AE.

### AE did not cause apoptotic cell death in OVCAR3 and SW626 cells

**Figure 3:**
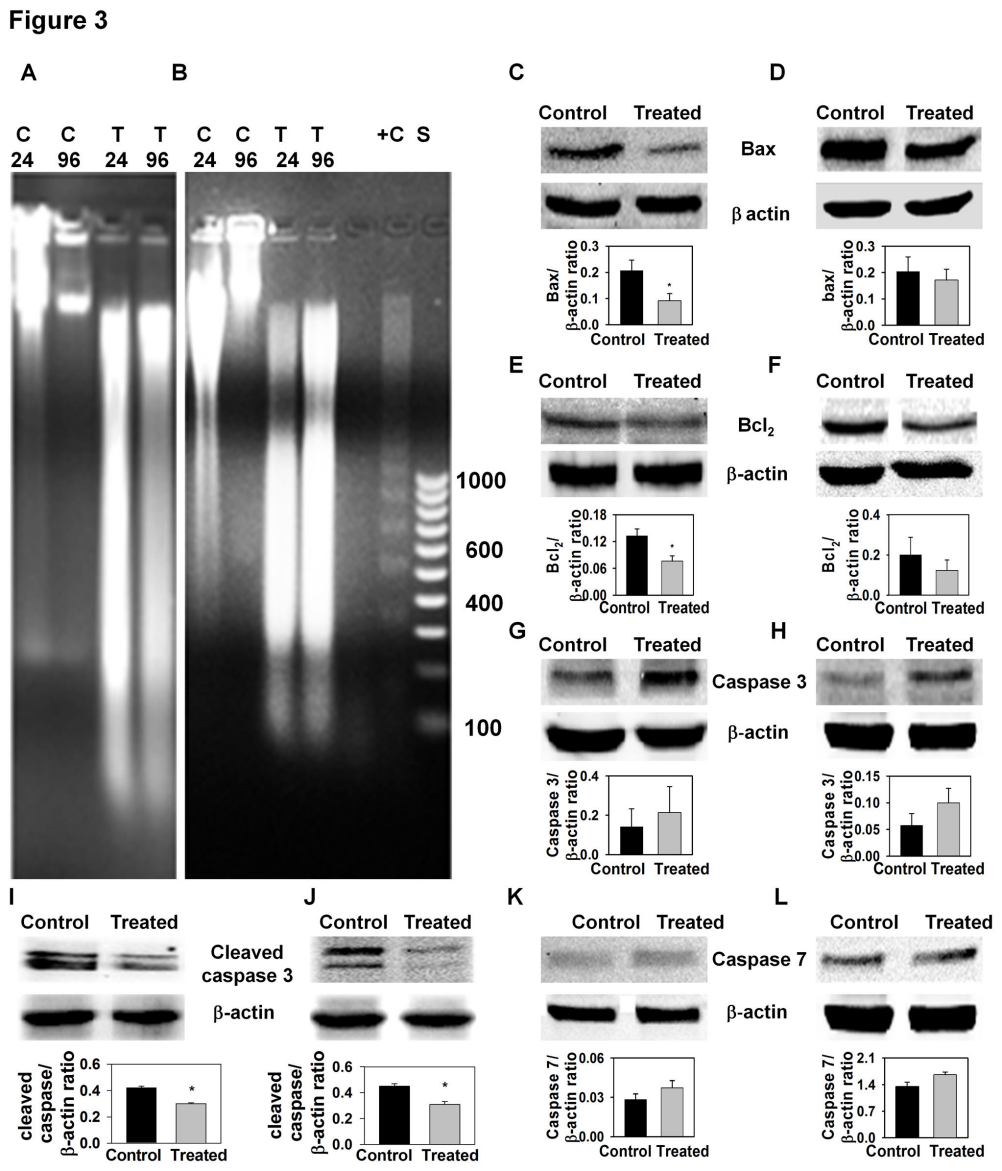
AE treatment does not cause apoptotic cell death. A–B. Representative photographs of DNA fragmentation in OVCAR3 cells (A) and SW626 cells (B). OVCAR3 and SW626 cells were treated with 300 µg/ml AE as described in Materials and Methods. After treatment, DNA was extracted and electrophoresed on 1% agarose gel. The size in base pairs of the molecular weight markers (lane) is indicated alongside the gel. The number on the right side indicates the length in nucleotides for several of the bands. C24 = Control 24 hour, C96=Control 96 hour, T24=AE 24 hour treatment, T96=AE 96 hour treatment, +C=Positive control, S=DNA size standard. C–L. Expressions of apoptotic proteins after AE treatment (300 µg/ml) in OVCAR3 and SW626 cells. Representative photographs of Western blot of Bax (C, D), Bcl_2_ (E, F), Caspase 3 (G, H), cleaved Caspase 3 (I, J), Caspase 7 (K, L) in OVCAR3 (C, E, G, I, K) and SW626 (D, F, H, J, L) cells after AE treatment are shown on the top. β-actin was detected as a control for each blot. The histogram corresponding to each photograph represents the ratio of densitometric analysis of each band to the respective β-actin band. The culture was treated with 0 or 300 µg/ml AE for 24 hour. After treatment, protein from OVCAR3 and SW626 cells was extracted and 30 µg proteins were electrophoresed on SDS-PAGE. The protein was then transferred to nitrocellulose membrane and immunoblotted against apoptosis related antibodies - Bax, Bcl_2_, Caspase 3, cleaved Caspase 3 or Caspase 7. The values are means + S.E.M. of 4 independent experiments. *, p<0.05, as compared with control group.

In view of the morphologic changes described above, suggesting that treatment of OC cell lines with 300 µg/ml of AE induced cell death, we wanted to determine whether AE treatment induced apoptosis of these cell lines. We used two different approaches to explore this possibility: 1) Examination of DNA for internucleosomal fragmentation, a hallmark of apoptotic cell death, and; 2) Western blotting for specific proteins (e.g. bax, bcl_2_, caspase 3, cleaved caspase 3 and caspase 7) involved in the apoptotic pathway. To determine if AE caused DNA internucleosomal fragmentation, DNA prepared from control and AE-treated cultures was fractionated on 1% agarose gels 24 and 96 hours after the addition of AE (300 µg/ml). Although DNA degradation was observed in these preparations, there was no apparent DNA fragmentation even after 96 hours of treatment (Figure 3A-B), suggesting that cell death did not occur by apoptosis. To further explore the potential role of apoptotic pathways in death of AE treated cells, Western blots for bax, bcl_2_, caspase 3, cleaved caspase 3 and caspase 7 were performed. In OVCAR3 cells, AE treatment reduced the expression of bax, bcl_2_ and cleaved caspase 3 (Figure 3C, 3E and 3I), and did not change the expression of caspase 3 or caspase 7 proteins (Figure 3G and 3K). In SW626 cells, AE treatment did not change the expression of bax, bcl_2_, caspase3, or caspase7 (Figures 3D, 3F, 3H and 3L) and reduced the expression of cleaved caspase 3 (Figure 3J). Collectively, the results support the conclusion that apopotic pathways are not activated in AE treated OC cell lines.

### AE induces autophagy in OVCAR3 and SW626 cells

**Figure 4:**
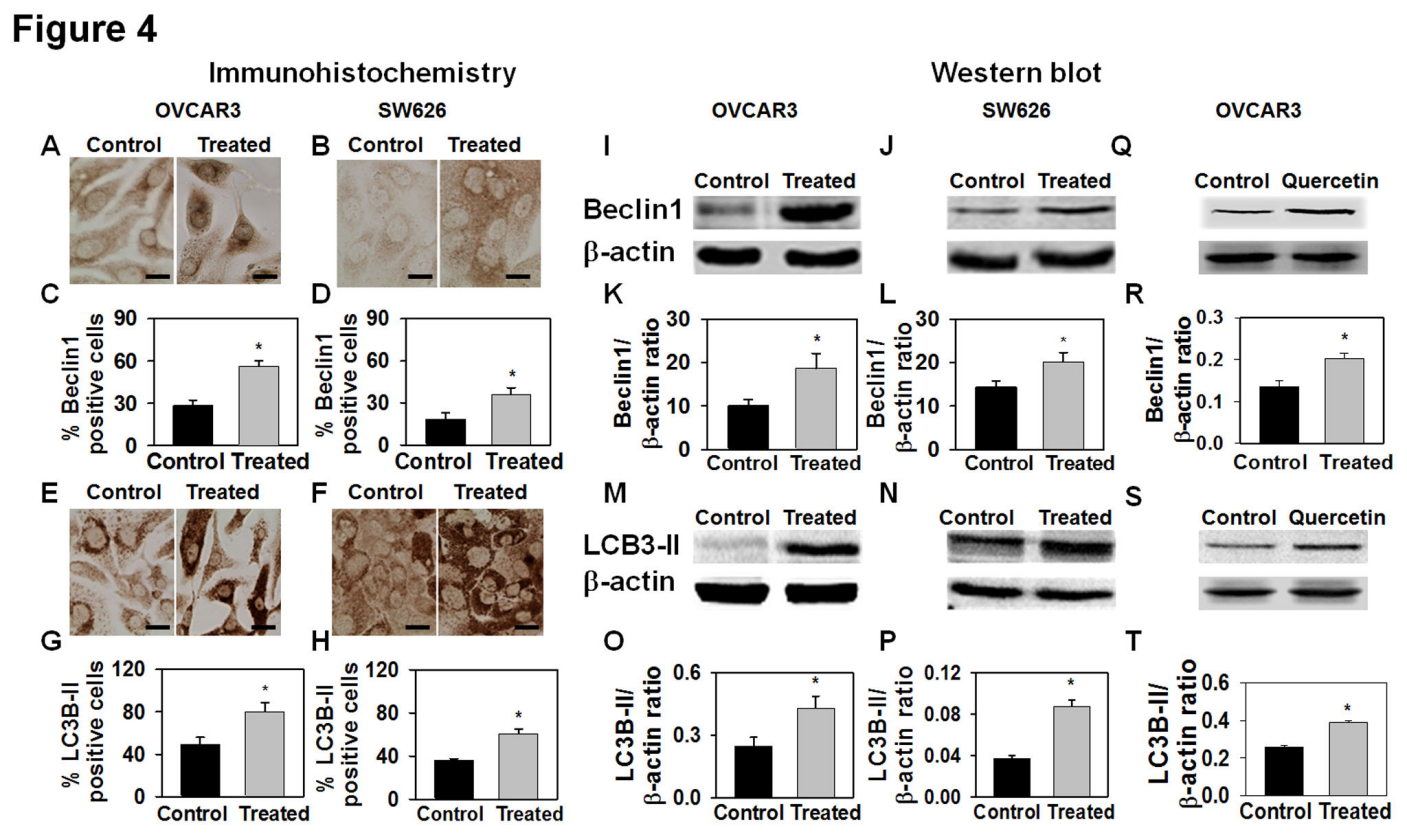
AE treatment increases beclin1 and LC3B-II expression in OVCAR3 and SW626 cells. Immunostaining of beclin1 and LC3B-II in OVCAR3 and SW626 control cells and after being treated with 300 µg/ml AE (A, B, E and F). The histograms show the percentage of beclin1 (C and D) and LC3B-II (G and H) immunopositive cells as a percentage of total cells. OVCAR3 and SW626 cells were cultured and grown for 2 days in DMEM in the presence of 10% serum as described under Materials and Methods. After this period, the cultures were fed with medium containing 10% serum and AE for 24 hours. Cells were photographed at 400 x magnification. Expression of beclin1 in total protein of OVCAR3 (I) and SW626 (J) cells and expression of LC3B-II in total protein of OVCAR3 (M) and SW626 (N) cells after being treated with AE (300 µg/ml) for 24 hrs. Representative photograph of western blot for beclin1 and LC3B-II are shown on the top. β-actin was detected as control for each blot. Mean + S.E.M. values of densitometric ratio of beclin1 (K and L) and LC3B-II (O and P) with β-actin are shown on the bottom of each respective gel. Q–T: Quercetin treatment (5 µg/ml for 48 hour) induces expression of beclin1 (Q, R) and LC3B-II (S, T) in OVCAR3 cells. After treatment, protein from OVCAR3 and SW626 cells was extracted and 50 µg proteins were electrophoresed on SDS-PAGE. The protein was then transferred to a nitrocellulose membrane and immunoblotted against beclin1 and LC3B-II antibodies. After immunodetection, beclin1 and LC3B-II positive bands were measured densitometrically and normalized with β-actin values. The values are means + S.E.M. of 6 independent experiments. *, p<0.05, as compared with control group. Bar=50 µm.

Recent *in vivo* and *in vitro* evidence suggests that a number of anticancer drugs exert their effects, at least partially, through their effects on autophagy [22,23]. The studies above suggested that apopotic pathways were not activated by AE treatment, so we opted to determine whether autophagy was activated in AE treated OC cells. Specifically, we treated OVCAR3 and SW626 cells with AE to determine the effects on expression of autophagic proteins- beclin1 and LC3B-II using immunocytochemistry and immunoblotting techniques. Immunoreactivity for both beclin1 and LC3B-II was present in untreated OVCAR3 and SW626 cells (Figure 4 A, B, E, F). The intensity of immunostaining and number of immunopositive cells, however, were increased by AE treatment [Figure 4 A–H]. Western blot analysis was consistent with results of immunostaining, demonstrating that AE treatment increased the expression of beclin1 and LC3B-II in both OVCAR3 and SW626 cells (Figure 4 I–P). These data suggest that autophagy is activated in AE treated cells. We also studied expression of the autophagic proteins beclin1 and LC3B-II after quercetin treatment (5 µg/ml for 48 hour) in OVCAR3 cells using immunoblotting. Expression of beclin1 and LC3B-II was significantly higher in quercetin treated cells than control (Figure 4 Q–T).

### AE inhibits angiogenesis-related genes in OVCAR3 cells

**Figure 5:**
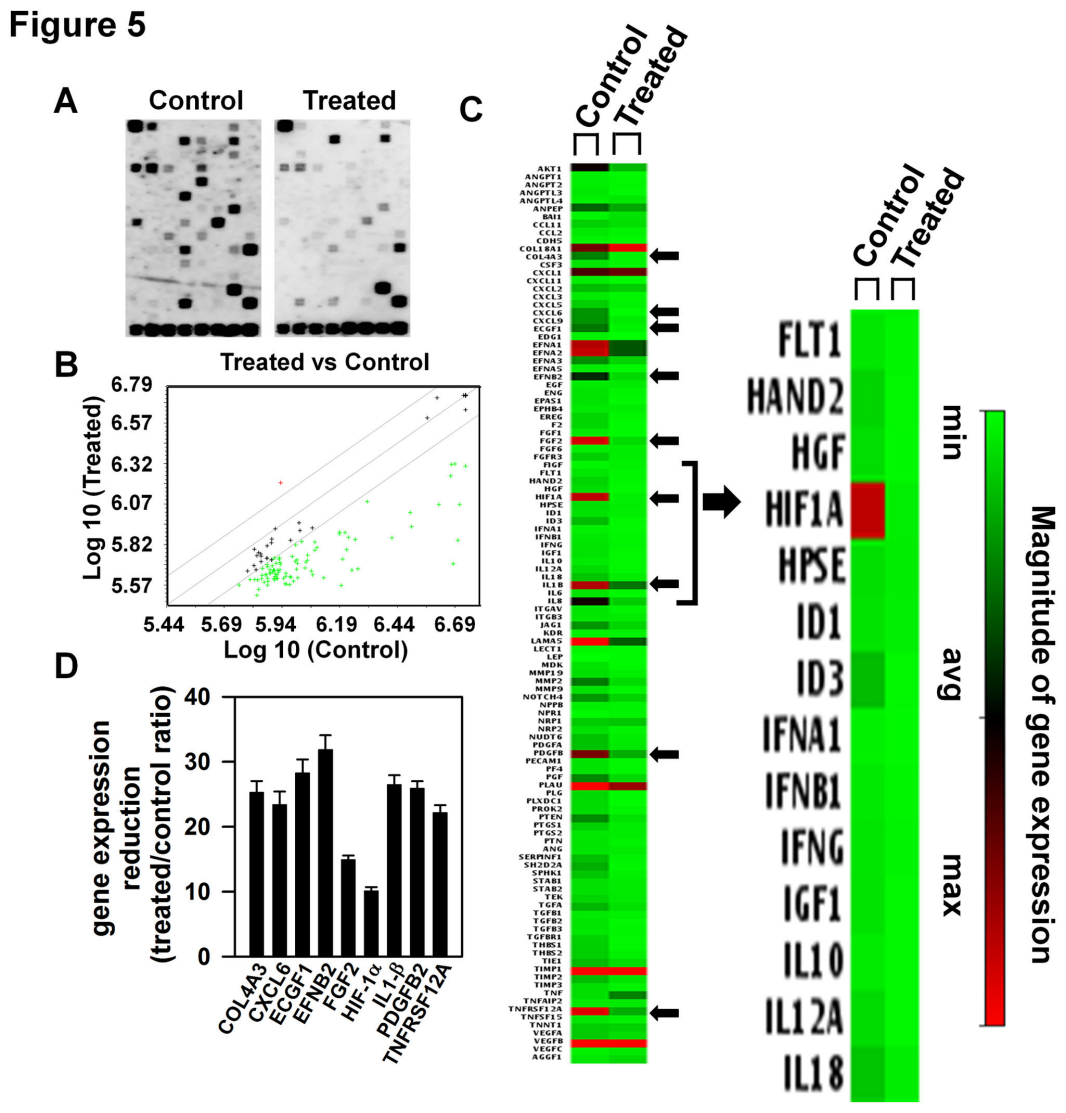
AE treatment reduces expression of angiogenesis related genes in OVCAR3 cells. A. The representative photographs of micro array from control and AE-treated culture. The culture was treated with 0 or 300 µg/ml AE for 24 h. After treatment, RNA was isolated using trizol extraction method. The superarray membranes were hybridized with biotin labeled cDNA, incubated with alkaline phosphatase-conjugated streptavidin, the gene expression was detected with the chemiluminescent substrate CDP-Star. B. Representative scatterplot of AE-treated vs control cell cultures. Many genes (green _*_) are under expressed in AE-treated group. C. Left panel: Heat map (clustergram) of control and AE-treated cultures. Nine genes which are reduced by more than 70% are indicated by arrowheads on the right side of the heat map. Middle panel: An enlarged heat map showing reduced expression of Hif-1α in the AE-treated group. Right panel: The magnitude of gene expression. D. Graphic representation of the relative gene expression using global background and GAPDH as reference gene and converted to fold-change values (AE versus control).

In view of the fact that a growing tumor mass must establish a vascular supply, and recent reports that angiogenesis could be inhibited by herbal remedies that included AE [8], we wanted to determine whether AE could inhibit angiogenesis *in vitro*. Specifically, we studied the effects of AE treatment on the expression of angiogenic genes in AE-treated (300 µg/ml) and untreated OVCAR3 cells using the Human Angiogenic OligoGE Array which detects 112 genes specifically involved in angiogenesis (SA Bioscience Corporation). The experiments were performed in three independent studies and the results are shown in Figure 5 A–D. Many genes (green _*_) were expressed at reduced levels in the AE-treated cells as compared to untreated controls (Figure 5B). A clustergram depicting the results obtained in control and AE-treated cultures is shown in Figure 5C. The expression of COL4A3, CXCL6, ECGF1, EFNB2, FGF2, IL1β, PDGFB, TNFRSF12A and HIF-1α were reduced to less than 40% of control levels (Figure 5D), with Hif-1α being inhibited to the greatest extent.

### AE inhibits expression of HIF-1α in vitro

**Figure 6:**
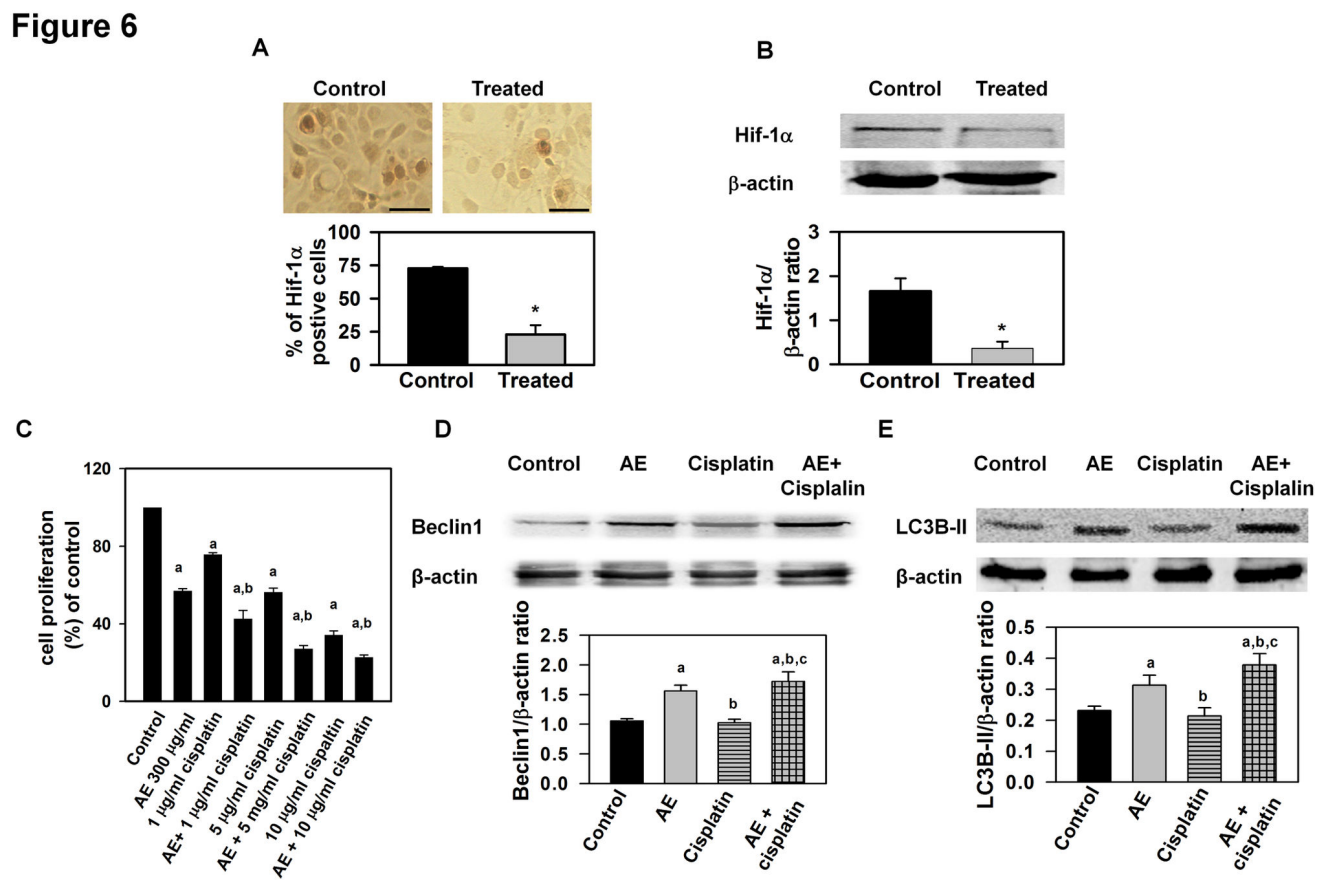
AE treatment reduces the expression of Hif-1α in OVCAR3 cells *in vitro*, and with cisplatin synergistically reduces cell proliferation and induces autophagy *in vitro*. A. Photomicrograph showing reduced expression of Hif-1α immunostatining in OVCAR3 cells after AE treatment. The histogram shows the percentage of Hif-1α immunopositive cells compared to total cells. OVCAR3 cells were cultured and grown and treated with 0 or 300 µg/ml of AE for 24 hours. Cells were immunostained with Hif-1α antibody and photographed at 400X magnification. Bar=50 µm. The values are means + S.E.M. of 4 independent experiments. *, p<0.05, as compared with control group. B. A representative photograph of Western blot for Hif-1α is shown on the top. β-actin was detected as a control for each blot. Mean + S.E.M. values of densitometric ratio of Hif-1α and β-actin are shown on the bottom of the gel. After immunodetection, the volume of Hif-1α positive bands was measured densitometrically and normalized with β-actin values. The values are means + S.E.M. of 4 independent experiments. *, p<0.05, as compared with control group. **C**. The histogram shows the synergistic effect of AE in presence of different doses of cisplatin on cell proliferation in OVCAR3 cells. OVCAR3 cells were cultured and grown for 2 days in DMEM in presence of 10% serum as described under Materials and Methods. After this period, the cultures were fed with medium containing 10% serum and 300 µg/ml AE with or without different doses of cisplatin for 24 hours. Data are the mean + S.E.M. from 6 independent observations. a, p<0.05, significantly different from vehicle-treated control group, b, p<0.05, significantly different from cisplatin group. D and E. The synergistic effect of AE with cisplatin (5 µg/ml) on autophagy. D. Representative photograph of a Western blot showing increased expression of beclin1 in AE, cisplatin and AE with cisplatin treated groups. E. Representative photograph of a Western blot showing increased expression of LC3B-II in AE, cisplatin and AE with cisplatin treated groups. OVCAR3 cells were treated with 0, AE (300 µg/ml), cisplatin (5 µg/ml) and AE (300 µg/ml) with cisplatin (5 µg/ml) for 24 hours. β-actin was used as a control for each blot. Mean + S.E.M. values of densitometric ratio of respective protein (beclin1 and LC3B-II) and β-actin are shown on the bottom of each gel. a, p<0.05, significantly different from vehicle-treated control group; b, p<0.05, significantly different from AE group; c, p<0.05, significantly different from cisplatin group.

In the above experiments, we demonstrated that AE treatment suppressed expression of numerous genes associated with angiogenesis. In order to determine whether decreased gene expression corresponded to decreased protein levels, we specifically examined the effect of AE treatment on production of Hif-1α protein in OVCAR3 cells using immunocytochemistry and Western blots. Both immunocytochemistry and Western blot results showed significantly reduced expression of Hif-1α in OVCAR3 cell cultures (Figure 6 A-B), further supporting the concept that AE treatment suppresses angiogenesis.

### AE with cisplatin synergistically reduced cell proliferation in OVCAR3 cells

In proliferation experiments, we demonstrated that AE reduced OVCAR3 cell proliferation in a time and dose dependent manner. We next sought to determine whether AE with cisplatin (a first line chemotherapeutic drug for OC) could synergistically reduce cell proliferation in OVCAR3 cells. We treated OVCAR3 cells with AE (300 µg/ml) in combination with cisplatin (1-10 µg/ml for 24 hour). We found that AE with cisplatin synergistically reduced cell proliferation in OVCAR3 cells (Figure 6C).

### AE with cisplatin synergistically induced autophagy in OVCAR3 cells

We also studied whether AE and cisplatin could work synergistically to induce cell death via autophagy. We treated OVCAR3 cells with AE (300 µg/ml) with cisplatin (5 µg/ml) for 24 hour. We found that AE with cisplatin synergistically induced expression of the autophagic proteins beclin1 and LC3B-II in OVCAR3 cell (Figure 6 D-E).

### AE inhibits growth and angiogenesis, and induces autophagy in tumors in athymic (nude) mice

**Figure 7:**
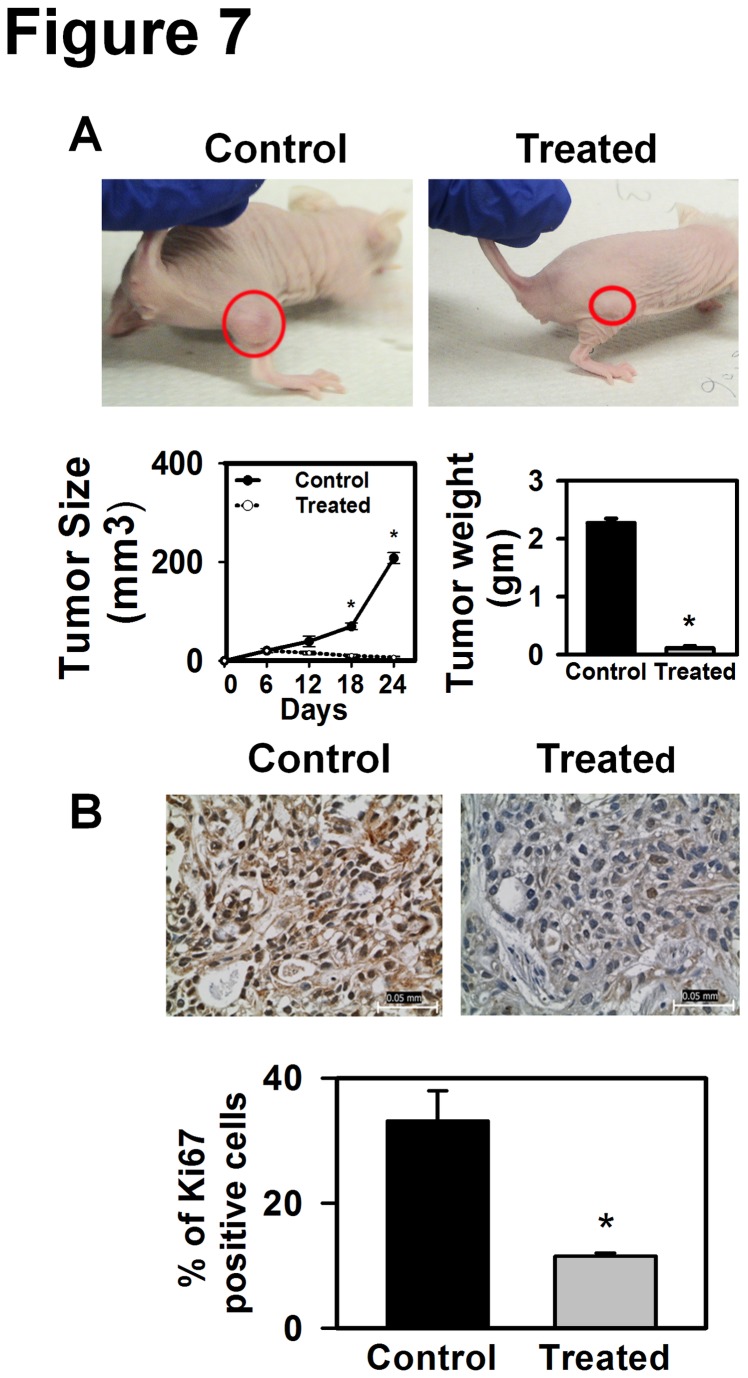
AE treatment inhibits growth of xenografted tumors *in vivo*. A. Top: Nude mice bearing tumors, left – control, right – treated. Bottom left. Size of tumors. Bottom right. Wet weight of tumor at 24 days of injection. B. Decreased immunohistochemical expression of Ki67 positive cells in mouse tumor xenograft after AE treatment. Histogram showing the decreased percentage of Ki67 immunopositive cells in AE-treated tumors. The tissue sections were photographed at 400X magnification. Bar=50 µm. The values are means + S.E.M. of 5 different mice. *, p<0.05, as compared with control group.

To determine whether AE treatment could reduce tumor growth and induce autophagy *in vivo*, OVCAR3 cells were injected into the right flank of nude mice. Within 6 days of inoculation, tumors grew to form visible masses. At this time (i.e. 6 days after inoculation) animals were divided into a non-treated control group, and a treated group (N=5 mice/group). The non-treated control group was fed 10% sucrose solution, whereas the treated group received AE (100 mg/kg body weight/day in 10% sucrose) for 18 days. Figure 7A shows that tumors grew more slowly in AE treated vs. control mice, with significant differences in size at 18 days of treatment and 24 days after inoculation (p=0.005). Mice were sacrificed 24 days after inoculation and the tumors were excised and weighed. Tumor mass was significantly reduced in AE treated vs. control mice (Figure 7A). To determine whether AE treatment could reduce cell proliferation in xenograft tumors similar to cells in culture, we detected Ki67 (a cellular marker for proliferation [24]) in control and AE treated xenograft tumor sections. AE treatment significantly reduced (p=0.006) the number of Ki67 positive cells (Figure 7B). These results suggest that AE treatment reduced cell proliferation in xenograft tumors. There also appeared to be no obvious toxicities associated with AE treatment as demonstrated by no apparent changes in liver, spleen (gross morphology), body weight or behavior (food and water intake, movement) of AE treated animals vs. controls (data not shown). These data indicate that AE is a potentially effective therapeutic agent for treating OC with no obvious toxicity in mice.

**Figure 8:**
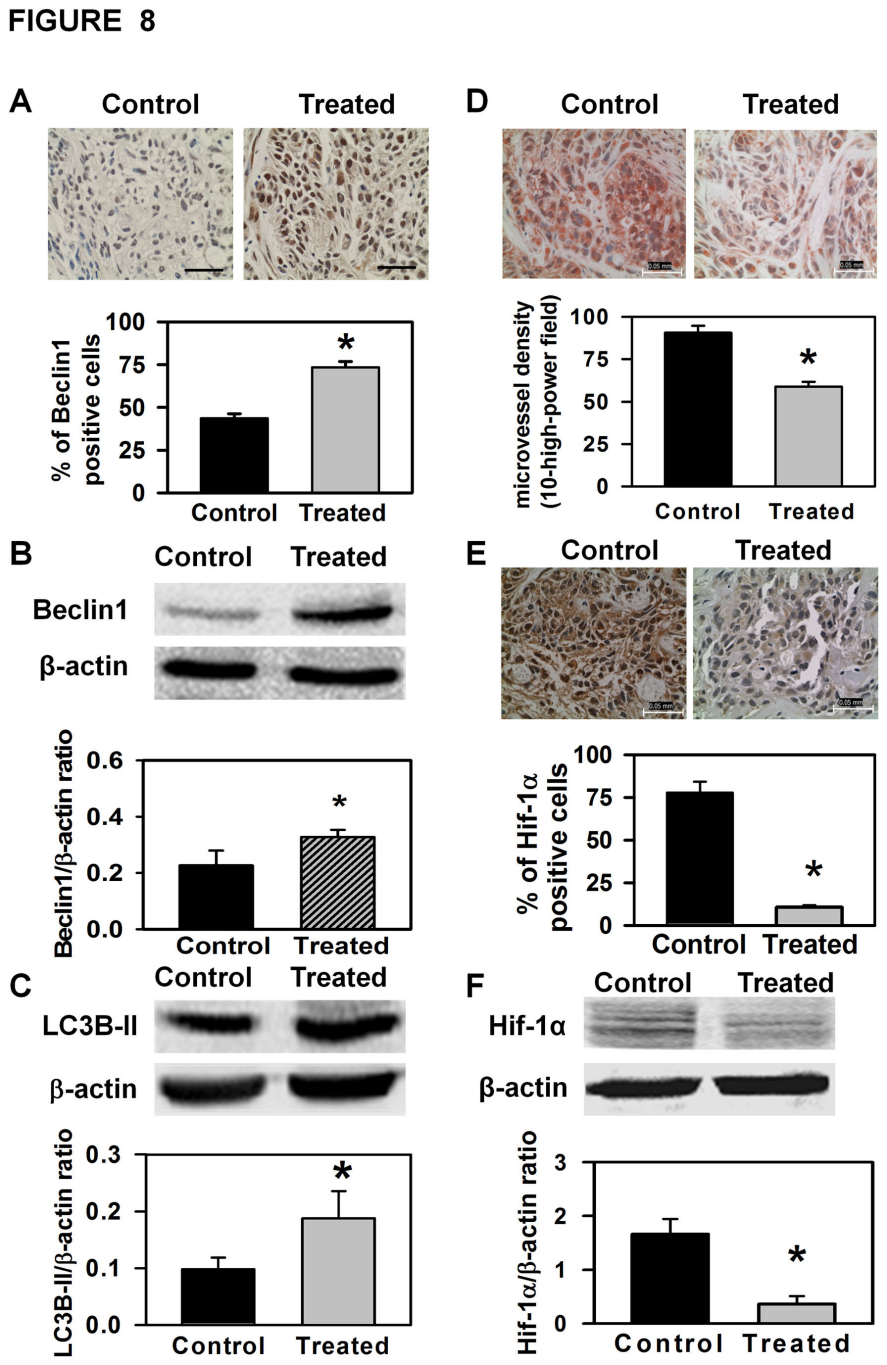
AE treatment induces autophagy, and inhibits angiogenesis and Hif-1α expression in xenografted tumors. A. Photograph showing increased immunoexpression of beclin1 in xenografted tumors after being treated with AE. The histogram shows the percentage of beclin1 immunopositive cells compared to total cells in control and AE treated groups. B. Representative photograph of a Western blot showing increased expression of beclin1 in AE treated group. C. Representative photograph of a Western blot showing increased expression of LC3B-II in AE treated group. β-actin was used as a control for each blot. Mean + S.E.M. values of densitometric ratio of respective protein (beclin1 and LC3B-II) and β-actin are shown on the bottom of each gel. D. AE treatment decreased immunohistochemical expression of CD31 positive cells in mouse xenograft tumors. Histogram showing decreased microvessel density in xenografts of AE-treated mice. E. Photomicrograph showing reduced expression of HIF-1α immunostaining in xenograft tumors after AE treatment. The histogram shows the percentage of Hif-1α immunopositive cells compared to total cells. F. Representative photograph of a Western blot for Hif-1α is shown on the top. β-actin was used as a control for each blot. Mean + S.E.M. values of densitometric ratio of Hif-1α and β-actin are shown on the bottom of the gel. After immunodetection, the density of Hif-1α positive bands was measured and normalized with β-actin values. Tissue sections were photographed at 400X magnification. The values are means + S.E.M. of 5 different mice. *, p<0.05, as compared with control group. Bar=50 µm.

In the experiments described above, we demonstrated that AE treatment of OC cell lines increased expression of beclin1 and LC3B-II proteins, indicating that autophagy was activated by AE treatment in these cell lines. To determine whether comparable effects would occur *in vivo*, xenografts from AE treated mice and controls were examined for the presence of these autophagy associated proteins. First we stained histologic sections of xenograft tumors with antibody to beclin1 and demonstrated that beclin1 expression was markedly enhanced in xenografts from AE treated mice compared to controls (Figure 8A). Next, we extracted proteins from xenograft tumors and demonstrated, by Western blot analysis, that expression of both beclin1 and LC3B-II was increased in xenograft tumors from AE treated mice compared to controls (Figure 8 B-C). These *in vivo* results demonstrate AE treatment activates autophagy in xenograft ovarian tumors, which is entirely consistent with our *in vitro* findings.

In the experiments presented above, we demonstrated that AE treatment suppresses expression of a variety of genes associated with angiogenesis *in vitro*, and suppresses production of Hif-1α protein in OVCAR3 cells. Consequently, it was important to determine whether AE treatment had a similar effect on angiogenesis *in vivo*. First, we determined the effect of AE treatment on tumor vasularization by staining with CD31, which is used primarily to demonstrate the presence of endothelial cells in histological tissue sections. As demonstrated in Figure 8D, CD31 staining was reduced in xenograft tumors from AE treated mice as compared to controls. We also calculated microvessel density within the xenografts and found that it was significantly reduced (p=0.008) in xenografts from AE treated mice (Figure 8D). Finally, we utilized immunohistochemistry and Western blot analysis to examine xenografts from AE treated mice and control mice for the expression of the angiogenesis associated protein Hif-1α, and demonstrated that AE treatment reduces expression of Hif-1α (Figure 8 E-F). These collective results support the conclusion that AE treatment suppresses angiogenesis both *in vitro* and in OC xenografts.

## Discussion

The major findings of the current study are that treatment with AE inhibited proliferation of OC cell lines *in vitro*, and dramatically suppressed growth of OC xenografts in nude mice. The concentrations of AE used to inhibit growth of OC cell lines *in vitro* were not toxic for normal placental cells, and the doses of AE that were used to suppress growth of OC xenografts *in vivo*, did not have any obvious toxic effects. Examination of OC cell lines treated with AE failed to demonstrate DNA fragmentation, and Western Blot analysis failed to demonstrate an increase in proteins associated with apoptosis. Consequently, it does not appear that AE treatment triggered apoptotic pathways in OC cell lines. Examination of AE treated OC cell lines and OC xenografts, however, demonstrated increased expression of autophagy associated proteins. Thus, AE treatment was shown to up-regulate autophagy pathways in OC cells, both *in vitro* and *in vivo*. Several lines of evidence convincingly demonstrated that AE treatment inhibited angiogenesis in OC cell lines *in vitro* and in OC xenografts in vivo. AE treatment was shown to suppress expression of a number of genes associated with angiogenesis, and inhibited production of the angiogenesis associated protein, HIF-1α, in OVCAR3 cells *in vitro*. In OC xenografts, AE treatment was shown to significantly reduce expression of Hif-1α and the endothelial specific antigen CD31. Finally, microvessel density within xenografts was significantly reduced in AE treated mice. These collective results demonstrate that AE is a potentially effective therapeutic agent for treating OC that may inhibit tumor growth by activating authophagy and inhibiting angiogenesis.

AE has previously been shown to induce apoptosis in human osteoclasts [25]. Additionally, AE contains the bioactive compound - gallic acid which causes cell death via apoptotic pathway [26]. Thus, we initially hypothesized that the cells in our study were undergoing apoptosis with cytotoxic treatment of AE. However, we could not detect any DNA fragmentation (apoptosis) in the cells treated with the highest concentrations of AE, even after 96 h, although degradation of DNA was observed on agarose gels (Figure 3A, B). We further studied the expression of pro- and anti-apoptotic proteins after AE treatment in OC cells. Bcl-2 is an anti-apoptotic protein induced by a variety of physiologic and pathologic stimuli [27,28]. Bax is a pro-apoptotic protein and also counters anti-apoptotic effects of Bcl-2 [27,28]. It has been proposed that the ratio of Bax/Bcl-2 may govern the sensitivity of cells to apoptotic stimuli [29,30]. In this study, the expression of both Bax and Bcl2 was reduced by AE treatment, suggesting that AE treatment did not trigger apoptotic pathways in OC cells. Furthermore, Western blot data did not indicate altered caspase 3 expression. Caspase activation (cleavage of procaspase to active caspase) is unique and sensitive indicator of apoptosis [31]. Reduced cleaved caspase 3 expression after AE treatment confirmed that apoptotic pathways were not initiated by this treatment.

From our studies of apoptosis described above, we noted that AE treatment suppressed Bcl-2 levels in OC cells. Bcl-2 has been shown to inhibit beclin1 dependent autophagy [32], so we explored the possibility that AE treatment stimulated autophagy in OC cells. From our morphological studies, we noted multiple cytoplasmic vacuoles in AE treated OVCAR3 and SW626 cells and these morphologic changes are consistent with activation of the autophagic pathway since autophagy is characterized by accumulation of autophagic vacuoles in the cytoplasm [33–35]. Examination of AE treated OVCAR3 and SW626 cell lines and OC xenografts, demonstrated increased expression of the autophagy associated proteins, beclin1 and LC3B-II, by both immunostaining and Western Blot analysis, confirming that AE treatment activates autophagy in OC cells, both *in vitro* and *in vivo*.

Beclin1 is an autophagy related gene with disrupted expression in most human cancers [36]. Beclin1 is reported to be deleted in 40–75% of cases of human breast, ovarian, and prostate cancers [37]. Additionally, disruption of beclin1 function in mice results in decreased autophagy in lymphomas, lung carcinomas and mammary precancerous lesions [38]. Conversely, the induction of autophagy is a common property of many antineoplastic therapies suggesting autophagy is an important regulator of cancer cell death [39]. Consequently, the capacity of AE treatment to activate autophagy in OC cells may represent one critical mechanism by which it inhibits growth of OC cell lines *in vitro* and OC xenografts *in vivo.*

Angiogenesis is known to play a critical role in the growth and spread of cancer [40]. Multiple studies have shown that angiogenic factors secreted by tumor cells play a critical role in tumor angiogenesis [41–43]. Recent studies have shown that Triphala, an herbal remedy containing AE, reduced angiogenesis [8]. Consequently, we wanted to determine whether AE treatment inhibited angiogenesis. We demonstrated that AE treatment suppressed expression of a number of genes associated with angiogenesis in OVCAR3 cells using the Human Angiogenic OligoGE Array which detects 112 genes specifically involved in angiogenesis. We found multiple angiogenic factors are down regulated by AE treatment in OC cells. These include COL4A3, CXCL6, ECGF1, EFNB2, IL8, PDGFB, TNFRSF12A, FGF2 and Hif-1α [44–52]. Among these factors, Hif-1α is highly affected by AE treatment. Its expression is reduces by ~90% after treatment. Interestingly, multiple studies found Hif-1α is a key regulator of angiogenic factors including CXCL6, ECGF1, EFNB2, IL8, PDGFB, TNFRSF12A, FGF2 [50,51,53–58].

AE treatment also inhibited production of the angiogenesis associated protein, HIF-1α, in OVCAR3 cells *in vitro* as demonstrated by immunohistochemical staining and Western Blot analysis. In OC xenografts, AE treatment was shown to significantly reduce expression of the endothelial specific antigen CD31 by immunohistochemistry, and the angiogenesis associated protein Hif-1α by both immunohistochemistry and Western blot analysis. Finally, microvessel density within xenografts was significantly reduced in AE treated mice. These collective studies convincingly demonstrate that AE treatment suppresses angiogenesis in our *in vitro* and *in vivo* models of OC and antiangiogenic effect of AE may be mediated through the regulation of HIF-1α

In conclusion, this study indicates for the first time that AE is a naturally occurring plant extract that inhibits growth of OC cells *in vitro* and *in vivo*, perhaps through the activation of autophagy and inhibition of angiogenesis. AE could become a highly effective therapeutic agent for the treatment of OC in the future used either alone or in conjunction with currently used chemotherapeutic agents.
